# Neutrophil extracellular traps promote bronchopulmonary dysplasia-like injury in neonatal mice *via* the WNT/β-catenin pathway

**DOI:** 10.3389/fcimb.2023.1126516

**Published:** 2023-04-27

**Authors:** Liujuan Sun, Meiyu Zhang, Jin Jiang, Wanjiao Liu, Wenhao Zhao, Fang Li

**Affiliations:** ^1^ Ministry of Education Key Laboratory of Child Development and Disorders, Department of Neonatal Diagnosis and Treatment Centre Children’s Hospital of Chongqing Medical University, ChongQing, China; ^2^ National Clinical Research Center for Child Health and Disorders, ChongQing, China; ^3^ China International Science and Technology Cooperation base of Child Development and Critical Disorders, ChongQing, China; ^4^ Chongqing Key Laboratory of Pediatrics, ChongQing, China

**Keywords:** bronchopulmonary dysplasia, neutrophil extracellular traps, hyperoxia, bronchopulmonary dysplasia-like injury, Wnt/β-catenin pathway

## Abstract

**Background:**

Bronchopulmonary dysplasia (BPD) is one of the most common and severe chronic diseases in preterm infants. Premature infants are susceptible to BPD due to immature lungs and adverse perinatal episodes of infection, hyperoxia, and mechanical ventilation.

**Methods:**

Neutrophils are the first line of host defence, and the release of neutrophil extracellular traps (NETs) is an important strategy to immobilize and kill invading microorganisms. This study examined whether NETs were associated with BPD in preterm infants and contributed to hyperoxia-induced lung injury in neonatal mice *via* the WNT/β-catenin pathway.

**Results:**

In this study, we found that preterm infants with BPD had higher levels of NETs in their tracheal aspirates than those without BPD. Neonatal mice treated with NETs after birth exhibited BPD-like changes in their lungs. Furthermore, the levels of Aquaporin 5 (AQP5) and surfactant-associated protein C (SPC), which represent alveolar differentiation and development, were significantly lower than those in the controls. The WNT/β-catenin pathway is one of the most well-known signalling pathways involved in lung growth. We found that the expression of the target genes c-MYC, cyclin D, and vascular endothelial growth factor (VEGF) and the important proteins WNT3a and β-catenin significantly decreased. Moreover, heparin, which is a NET inhibitor, attenuated changes in gene and protein expression, thereby attenuating BPD-like changes.

**Discussion:**

This finding indicates that NETs are associated with BPD and can induce BPD-like changes in neonatal mice *via* the WNT/β-catenin pathway.

## 1 Introduction

Bronchopulmonary dysplasia (BPD) is a leading cause of chronic respiratory morbidity among survivors of preterm birth and is associated with the greatest risk for those born at 23–30 weeks. BPD in most premature infants is characterized by fewer but larger alveoli, mild fibrosis, and persistent inflammation that play key roles in its pathogenesis. Inflammation can be exacerbated by several perinatal factors, including mechanical ventilation, exposure to oxygen, and other sources of oxidants, such as parenteral nutrition and sepsis (Principi et al., 2018). According to recent research, innate immunity plays an important role in the development of BPD (Zaramella et al., 2019).

In 2004, Brinkmann first proposed that neutrophil extracellular traps (NETs) could be produced to clear extracellular pathogens, in addition to the traditional method of engulfment and degranulation. NETs are composed primarily of eDNA (free DNA), neutrophil elastase (NE), myeloperoxidase (MPO), histones, and granules (Brinkmann et al., 2004) and are involved in several inflammatory diseases of the lungs, such as chronic obstructive pulmonary disease (COPD) and acute lung injury (Jorch and Kubes, 2017; Söderberg and Segelmark, 2018). Sun et al. (2020) found that NETs were significantly increased in BPD rat models and that neutralizing antibodies and heparin could reduce lung damage by inhibiting NET formation. According to Davies et al. (2010) NETs in bronchoalveolar lavage were more active in premature infants with BPD than in those without BPD, suggesting that NETs might participate and play an important role in the pathogenesis of BPD (Xu et al., 2020).

According to Davies et al. (2010), NE in bronchoalveolar lavage is more active in preterm infants with chronic lung disease than in those without chronic lung disease. NE is an important component of NETs, and BPD is an important chronic lung disease. This finding suggests that NETs may be involved in the pathogenesis of BPD.

WNT/β-catenin signalling is essential for lung development (Zhang et al., 2012; Frank et al., 2016; Hussain et al., 2017) and was shown to be downregulated by NE, which is an important component of NETs in COPD and emphysema (Kneidinger et al., 2011). However, whether NETs can promote BPD *via* the WNT/β-catenin signalling pathway remains unclear. We examined the relationship between NETs with BPD and examined the effect of NETs on BPD *via* WNT/β-catenin.

## 2 Materials and methods

### 2.1 Tracheal aspirate collection and NETs measurement

Tracheal aspirates were collected through a tracheal catheter, added to 2 times the volume of 0.1% DDT solution, mixed for 15 seconds, diluted 1:1 with phosphate-buffered saline and then centrifuged at 3,000 × g for 15 min at 4°C. NE and MPO levels in tracheal aspirate samples were measured with an ELISA kit (Human Neutrophil Elastin ELISA Kit, Human MPO ELISA Kit) (Khan et al., 2019; Thanabalasuriar et al., 2019; Thierry and Roch, 2020). All experimental procedures were performed according to protocols approved by the Ethics Committee of Children’s Hospital Affiliated to Chongqing Medical University.

### 2.2 Induction, observation, and quantification of NETs

We collected peripheral venous blood from healthy adult volunteers with a heparin collection tube to extract neutrophils with a neutrophil kit (Dömer et al., 2021). All procedures were carried out in a sterile environment and at room temperature. To assess the induction of NETs, neutrophils were exposed to 500 nmol/L PMA (phorbol-12-myristate-13-acetate) (Thierry and Roch, 2020), and NETs were incubated at 37°C and 5% CO_2_ for 4 h. NETs formation was analysed by using the cell impermeable dsDNA dye SYTOX™ Green Nucleic Acid Stain (Invitrogen™). SYTOX Green kinetic analysis of NETs was performed using a Keyance BZ9000 Microscope (Osaka Keyance, Japan) with a 40× planar Fluor EL NA0.60 objective (Nikon, Tokyo, Japan). A total of 5×10^6^/L neutrophils in 100 μl and 20 μl of PMA were placed in an incubator with 5% CO_2_ at 37°C for 4 h. The concentration of eDNA was measured with Quant-iT PicoGreen and a double-stranded DNA Analysis Kit (Thermo P7589).

### 2.3 Animal models

#### 2.3.1 Determination of NETs in animal models

Wild-type C57BL/6J mice (female: 50 and male: 50) at 6–8 weeks of age were provided by the University Animal Center of Chongqing Medical Science. All mice were housed in a pathogen-free cage with ad libitum access to mouse chow and water and a 12 h light/dark cycle with constant temperature (25 ± 2°C) and 50% relative humidity. After natural birth, neonatal mice were used as animal models. All experimental procedures were performed according to protocols approved by the Experimental Animal Ethics Committee of Chongqing Medical University [wild-type C57BL/6J mice (female and male)].

Animal models were established as follows. In the hyperoxia-induced lung injury group, neonatal mice were exposed to hyperoxia (85% oxygen) in a sealed cage with continuous O_2_ monitoring to mimic BPD. Mice in the NET group were treated with 5 μl/g NETs by nasal drops every 3 days. The mice were treated with 5 μl/g NETs (0.43 ng/μl eDNA) by nasal drops every 3 days, injected with heparin (250 IU by intraperitoneal injection), and killed by cervical dislocation at 14 days (250 U/kg) (Chen et al., 2020; Sun et al., 2020) (Macklin H837056 200MG, 200MG). In the phosphate-buffered saline (PBS) control group, the mice were treated with 5 μl/g PBS by nasal drops every 3 days and exposed to air for 14 days until euthanasia was performed by injection of pentobarbital sodium (200 mg/kg i.p.).

#### 2.3.2 NE/MPO ELISA

Mouse lung tissue was ground, dissolved, and centrifuged, and the supernatant was collected for analysis by ELISA (Solarbio SEKM-0118-96T, elabscience E-EL-M3025) (Khan et al., 2019; Thanabalasuriar et al., 2019; Thierry and Roch, 2020).

#### 2.3.3 Immunofluorescence staining

We used airway instillation of 5 μl/g PBS for the negative control group. NETs (427.03 ng/ml, 5 μl/g) were administered by airway instillation once every 3 days for 14 days, at which time the lung tissue was collected for immunofluorescence analysis. We quantified NETs by immunofluorescence analysis and ‘NIS Element software. To assess NETs in lung tissue in each group, paraffin-embedded lung tissues were sectioned (4 µm), and the sections were fixed on glass slides. After allowing the tissue sections to stand at room temperature for 10 min, the paraffin sections were placed into xylene for 3 min, absolute ethanol for 5 min, 85% alcohol for 5 min, and 75% alcohol for 5 min before being washed with ddH2O for 5 min. We placed the tissue sections in a repair cassette filled with citric acid antigen retrieval solution (pH 6.0) and microwave antigen retrieval. After natural cooling, the slides were placed in PBS and washed 3 times for 5 min each with shaking on a decolorization shaker. After the slices dried, a circle was drawn around the tissue with a histochemical pen to prevent fluid loss. Endogenous peroxidase activity was inhibited using a 3% H2O2 solution for 15 minutes, and the specimens were blocked with PBS containing 1% bovine serum albumin (BSA, A8010, Solar). The blocking solution was gently dried, and the sections were incubated with anti-NE (Santa Cruz sc-365950) and anti-MPO (Abcam ab208670) at 4°C overnight. The slides were placed in PBS and washed 3 times on a decolorization shaker for 5 min each time. Then, the slides were incubated for 1 hour with Alexa Fluor 488 goat anti-mouse IgG1 (1070-02, Southern Biotech) and Alexa Fluor 546 donkey anti-rabbit (H+L) (Invitrogen A10040) secondary antibodies in the dark. The slides were again placed in PBS and washed 3 times on a decolorization shaker for 5 min each time. The sections were then stained with 4’,6-diamidino-2-phenylindole (DAPI, DA0004, Leagene Bioon).The slides were placed in PBS and washed 3 times for 5 min each with shaking on a decolorization shaker. When the slices had dried slightly, a small amount of anti-fluorescence quenching agent was dropped onto each slide, and the slide was sealed with resin mounting agent. We used a confocal microscope to visualize the slides. NETs were quantified by immunofluorescence analysis with ‘NIS Element’ software.

### 2.4 Lung haematoxylin and eosin staining

After the mice were euthanized by cervical dislocation and a sternotomy was performed, the lung was perfused transcardially with PBS at a pressure of 25 cm H_2_O to remove blood cells. The right bronchi were ligated with a string, and the lung was perfused through the trachea with 4% polyformaldehyde at a pressure of 20 cm H_2_O and fixed in 4% paraformaldehyde solution at 4°C for 24 h. The lung sections (4 mm) were stained with H&E to examine lung morphometry and determine the mean linear intercept (MLI) to assess lung development. The MLI was used to estimate the average diameter of a single alveolus by using the following formula: MLI = total length/alveolar septal number. The intercepts of the alveolar septal number were counted at the intersection point of the two lines, and the total length of all of lines combined were divided by the number of intercepts to provide the MLI of the region.

### 2.5 Quantitative real-time PCR

The total RNA was extracted from fresh lung tissue by using TRIzol reagent (LIFE 15596-026, Beijing, China) and reverse transcribed into cDNA with a reverse transcription kit (Evo M-MLV Mix Kit with DNA Clean for qPCR [Accurate Biology, AG11728, China)] according to the manufacturer’s instructions. Aquaporin 5 (AQP5), Surfactant-associated protein C (SPC), vascular endothelial growth factor (VEGF), c-MYC, cyclin D1, and GAPDH mRNA expression was quantified with a StepOnePlus Real-Time PCR System (ABI, USA). Primers with the following sequences (**Table 1**) were used:

**Table 1:** Primers for genes amplified by RT-PCR.

Gene	Primer sequence
Aqp5-F	CTTCCCCCAGGTAGACAGAG
Aqp5-R	AAACGCCCAACCCGAATAC
cyclinD1-F	AGTGTTGCTGGTGTGTGTTG
cyclinD1-R	CCATCTGAATGCGTGTGTGG
c-myc-F	AGTGTTCTCTGCCTCTGCCC
c-myc-R	TGGCTGTCGGGGTTTCCAA
VEGF-F	GGACTTGTGTTGGGAGGAGG
VEGF-R	CCAGGAATGGGTTTGTCGTG

The results were based on the 2−ΔCt method, and GAPDH was used as the internal control. Each sample was analysed in triplicate, and the results represent three independent experiments.

### 2.6 Western blotting

The relative levels of SPC, AQP5, β-catenin, and WNT3a in the lung tissues of mice in each group were determined by Western blotting. Total protein was obtained from the lung tissues by RIPA lysis buffer containing protease and phosphatase inhibitor cocktails (P0013B Beyotime, Shanghai, China). Protein concentrations were determined with a bicinchoninic acid (BCA) assay. The proteins were separated by 7.5, 10%, and 12.5% SDS−PAGE and transferred to polyvinylidene fluoride membranes (Solarbio, Beijing, China). The membranes were blocked for 1 h in blocking solution at room temperature and incubated at 4°C overnight with the following primary antibodies: anti-SFTPC (1:1500, Genetex GTX134340), anti-β-catenin (1:2000, Genetex GTX101435), rabbit anti-Wnt3a pAb (1:500, Zenbnio 822111), anti-AQP5 (1:1500, Genetex gex132400), and anti-β-actin (1:2000, Zenbio R23613). The membranes were then washed three times with Tris-buffered saline Tween-20 (TBST) and incubated with HRP-conjugated goat anti-rabbit IgG (1:2000, ZSBio, China) for 1 h at room temperature. β-Actin was used as an internal control. Western Chemiluminescent HRP Substrate (Millipore, MA, USA) was used, and the blots were visualized using the G: Box gel doc system (Syngene, UK).

### 2.7 Statistical analysis

In this study, SPSS 20.0 software or GraphPad Prism were used for statistical analysis and graph, and all data were statistically compared. The measured data are expressed as the mean ± standard deviation (x ± s), and independent sample t tests (n≥6) and nonparametric tests (n<6) were used to compare two groups. Differences with P<0.05 were statistically significant.

## 3 Results

### 3.1 NETs were increased in the tracheal aspirates of premature infants with BPD compared with those of premature infants without BPD

**Figure 1:**
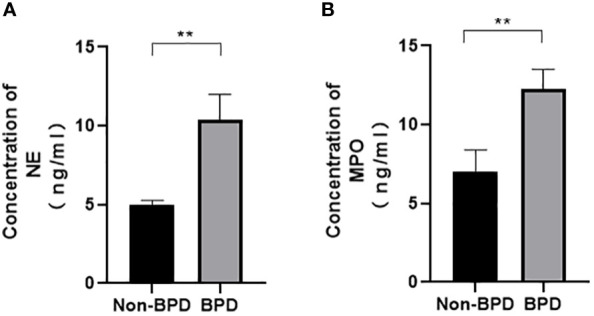
Comparison of NE/MPO levels in tracheal aspirates from patients with and without BPD. **(A)**. The level of NE in the tracheal aspirates of BPD and non-BPD patients. **(B)**. The level of MPO in the tracheal aspirates of BPD and non-BPD patients. Statistical analysis was performed by the Mann−Whitney test (n = 5). Asterisks indicate significant differences (**p ≤ 0.0079).

We hypothesized that NETs promoted the development of BPD. MPO and NE are the major components of NETs. We examined the levels of NETs in tracheal aspirates by ELISA. Samples were collected from 5 premature infants with BPD and 5 premature infants without BPD. We found higher levels of MPO and NE in the tracheal aspirates of preterm infants with BPD than in those of preterm infants without BPD (**Figure 1**).These results show that NETs concentrations differ amongst subjects with and without BPD and that the presence of BPD is strongly associated with NETs concentrations.

### 3.2 NETs were induced by PMA and quantified

**Figure 2:**
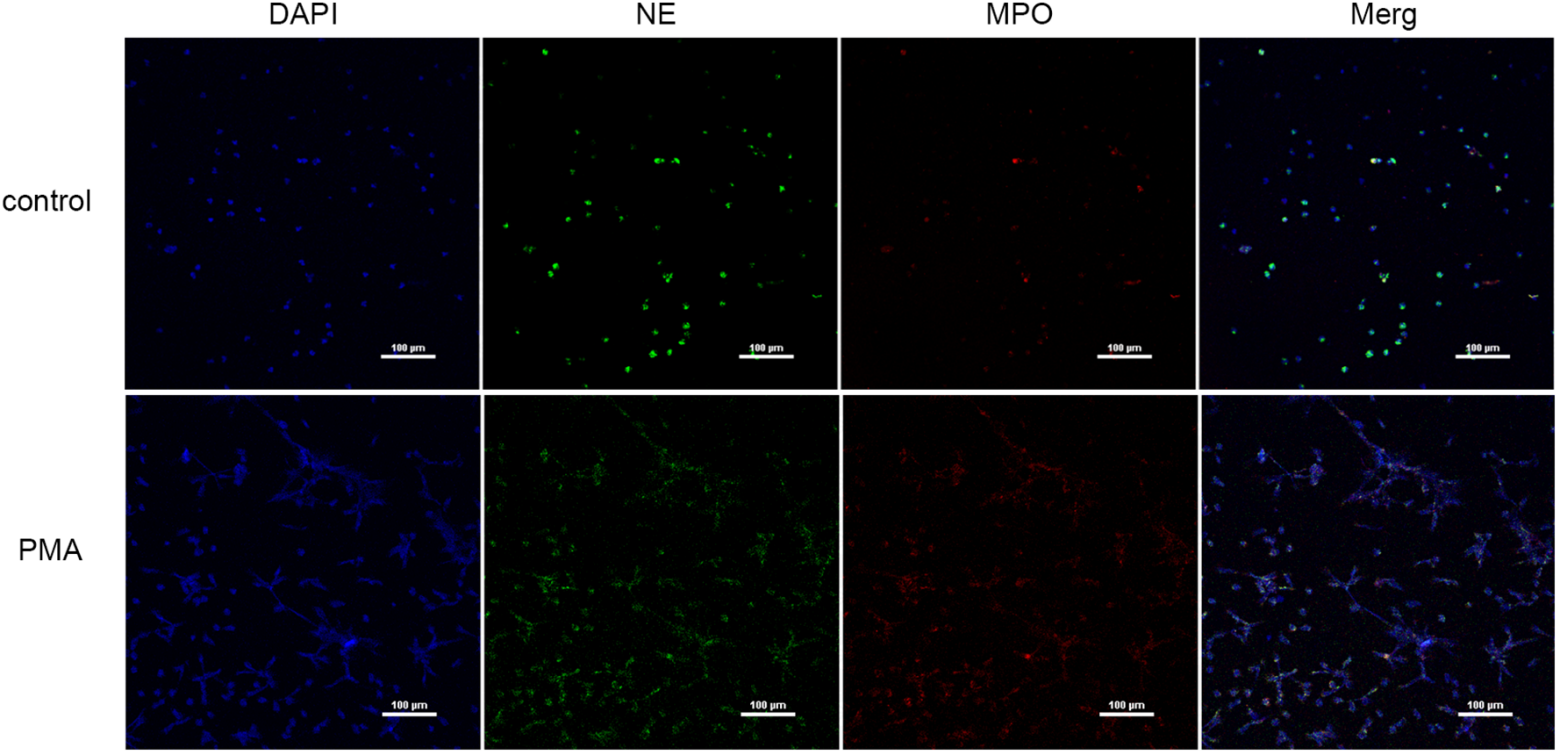
NETs were induced by PMA (immunofluorescence) (×200); bar: 100 µm.

We used PMA to induce peripheral blood neutrophils to produce NETs, and we qualitatively examined the presence of NETs and PBS by immunofluorescence (**Figure 2**) and measured eDNA levels with a microplate reader to calculate NET concentrations (**Table 2**). We found that PMA induced markedly greater production of NETs from neutrophils than PBS. In addition, the concentration of eDNA produced significantly decreased with decreasing PMA concentrations.

**Table 2:** eDNA concentration.

PMA	Concentration of PMN (cells/ml)	Concentration of NETs (ng/ml)	Concentration of PMN (cells/ml)	Concentration of NETs (ng/ml)
500 nmol/L	1× 10^7^	754.29	5× 10^6^	427.03
	1× 10^6^	133.39	5× 10^5^	47.25
	2× 10^5^	22.22	1× 10^5^	8.09
	1× 10^5^	13.09	5× 10^4^	3.13

### 3.3 Distribution and quantification of NETs in hyperoxia-induced lung injury

**Figure 3:**
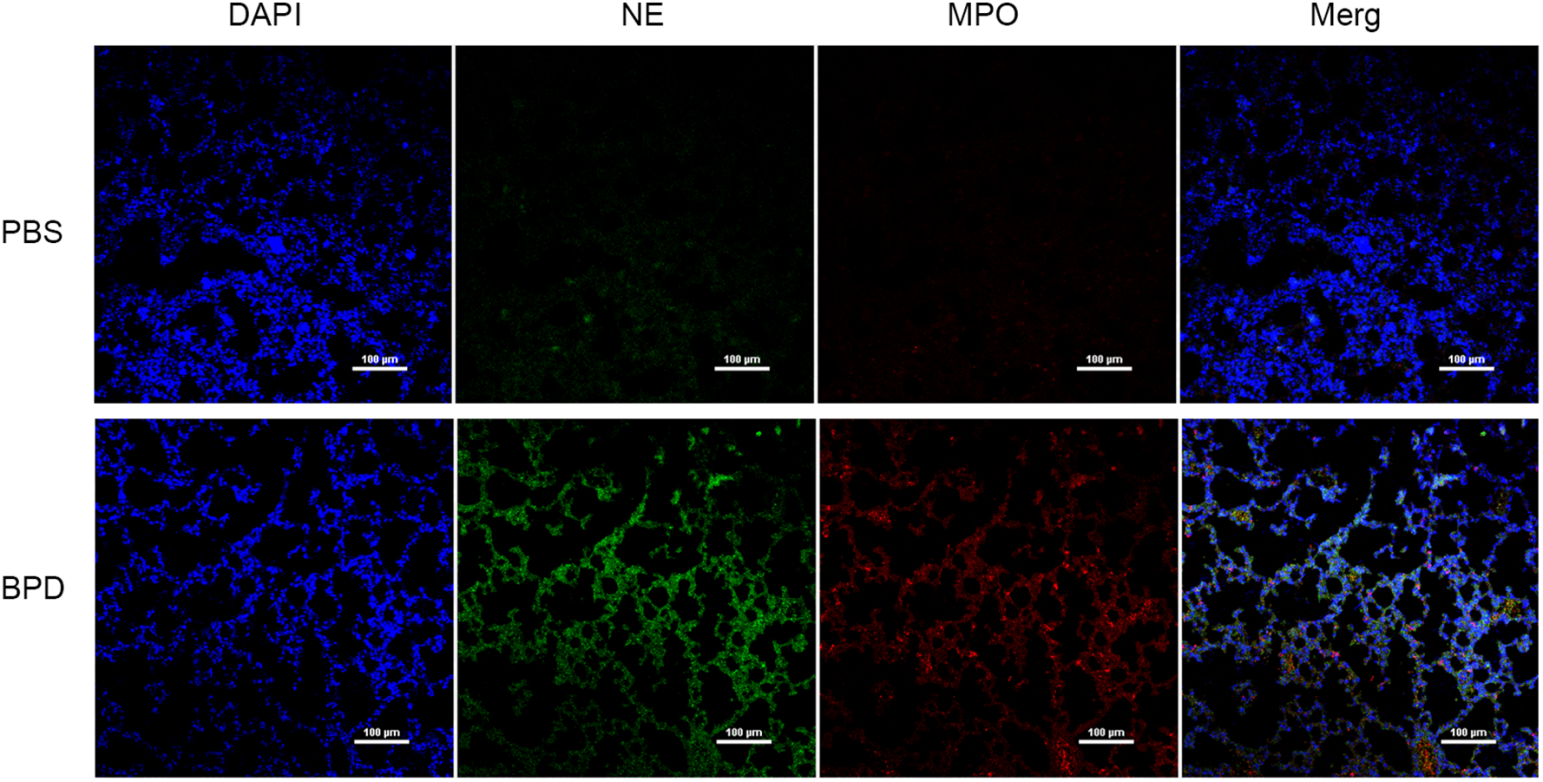
Comparison of the immunofluorescence of NETs in the PBS control group and hyperoxia-induced lung injury group (×200); bar: 100 µm.

**Figure 4:**
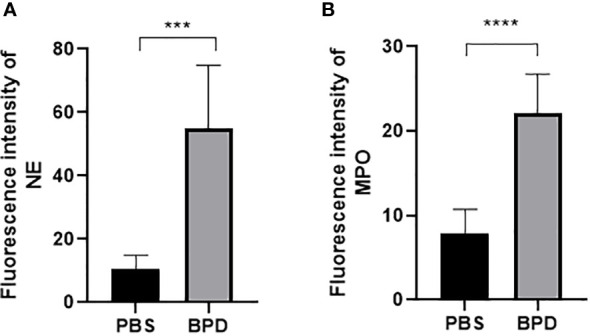
Fluorescence intensity. **(A, B)**. Fluorescence intensity of NE and MPO. Statistical analysis was performed by one-way ANOVA with a *post hoc* Tukey’s test (n=6, ***p ≤ 0,0003 ****p<0.0001).

**Figure 5:**
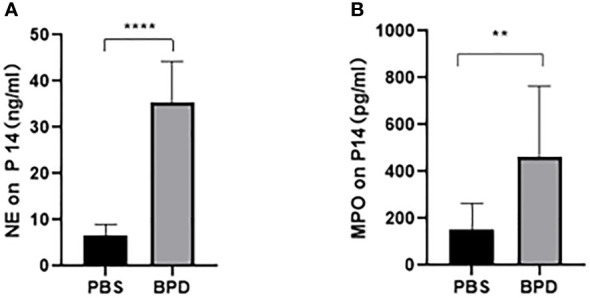
ELISA analysis of NETs in the PBS control group and the hyperoxia-induced lung injury group. **(A, B)**. Comparison of NE and MPO levels in the PBS control group and the hyperoxia-induced lung injury group. Statistical analysis was performed by unpaired t test. Asterisks indicate significant differences (n=10, ****p ≤ 0.0001 **p ≤ 0.003 unpaired t test).

Immunofluorescence analysis (**Figures 3**, **4**) and ELISA (**Figure 5**) showed that lung levels of MPO and NE in the hyperoxia-induced lung injury group were significantly higher than those in the PBS control group. This illustrated that the hyperoxia-induced BPD group had a higher content of NETs, which was consistent with the clinical results. The animal trials also demonstrated a close association between NETs and BPD.

### 3.4 NETs can cause BPD-like changes in preterm mice

**Figure 6:**
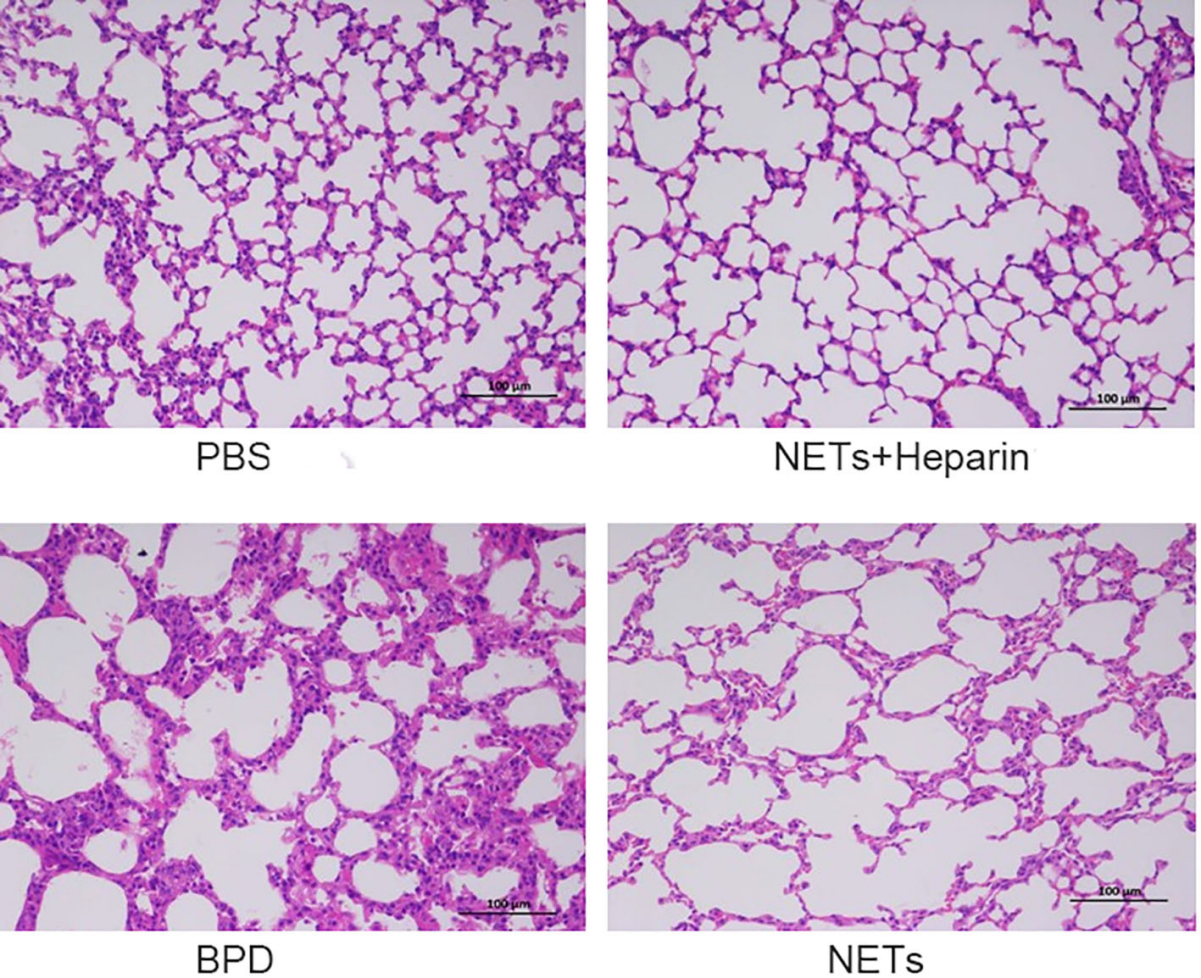
Effect of NETs on the lungs over 14 days. H&E assessment of lung tissue in each group (×200).

**Figure 7:**
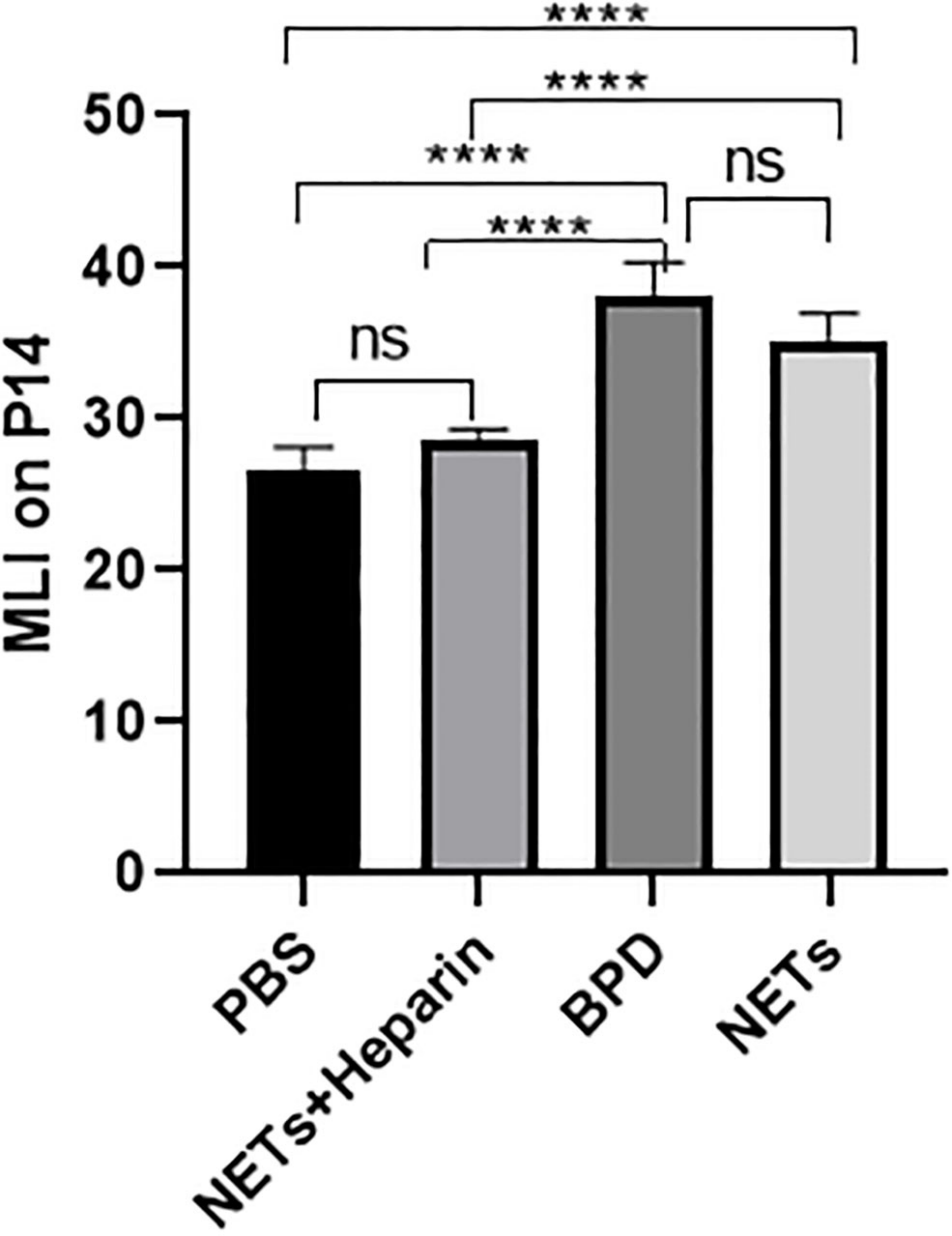
Comparison of MLI values for each group. Comparison of MLI values for each group. Statistical analysis was performed by ordinary one-way ANOVA with a *post hoc* Tukey’s test. Asterisks above the bars indicate significance compared to untreated cells (n=11, ****p ≤ 0.0001; n.s., not significant, one-way ANOVA).

We examined the lungs in the NET group to determine whether NETs could cause BPD-like changes. In addition, the inhibitor heparin was administered to preterm mice in the NET group to determine whether BPD-like changes were alleviated by inhibitors. The mice in the NET group exhibited similar lung injuries as those in the hyperoxia-induced lung injury group, which were characterized by a widened alveolar septum and simplified alveolar developmental structure, as shown by H&E staining (**Figure 6**). The MLI was used to assess alveolar enlargement as a measure of impaired lung development. We compared the MLI between the PBS control group, NET group, and hyperoxia-induced lung injury group and found that it was significantly increased (P<0.05). Furthermore, lung injury was improved in the NET+heparin group compared to the NETs group and the hyperoxia-induced lung injury group (P<0.05 one-way ANOVA) (**Figure 7**).

### 3.5 NETs slow lung development

The levels of AQP5 and SPC are associated with alveolar epithelial cell differentiation and development. We examined the expression of AQP5 and SPC in lung tissue in each group to determine the effects of NETs on alveolar development.

**Figure 8:**
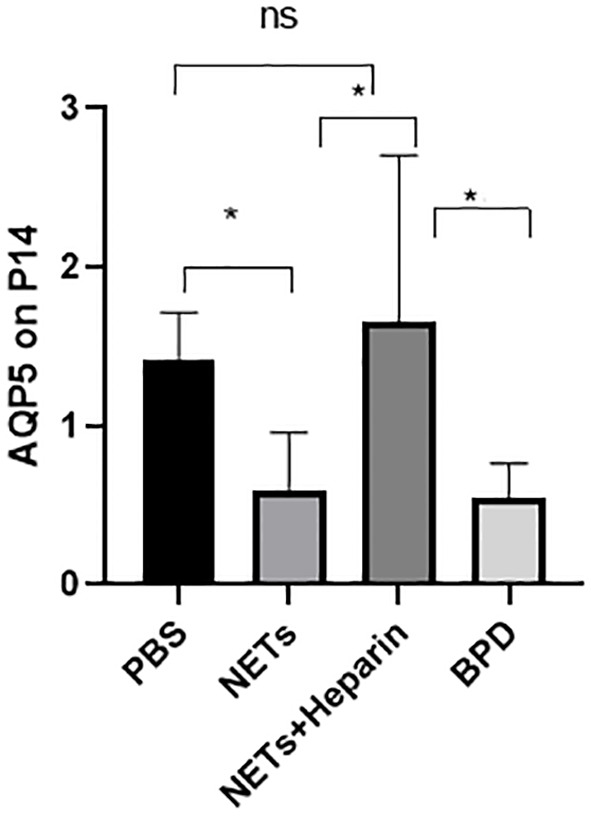
The effect of NETs on alveolar differentiation and development. AQP5 expression was determined by qPCR. Statistical analysis was performed by one-way ANOVA with a *post hoc* Tukey’s test (n=6, *p ≤ 0.001; n.s, not significant. one-way ANOVA).

**Figure 9:**
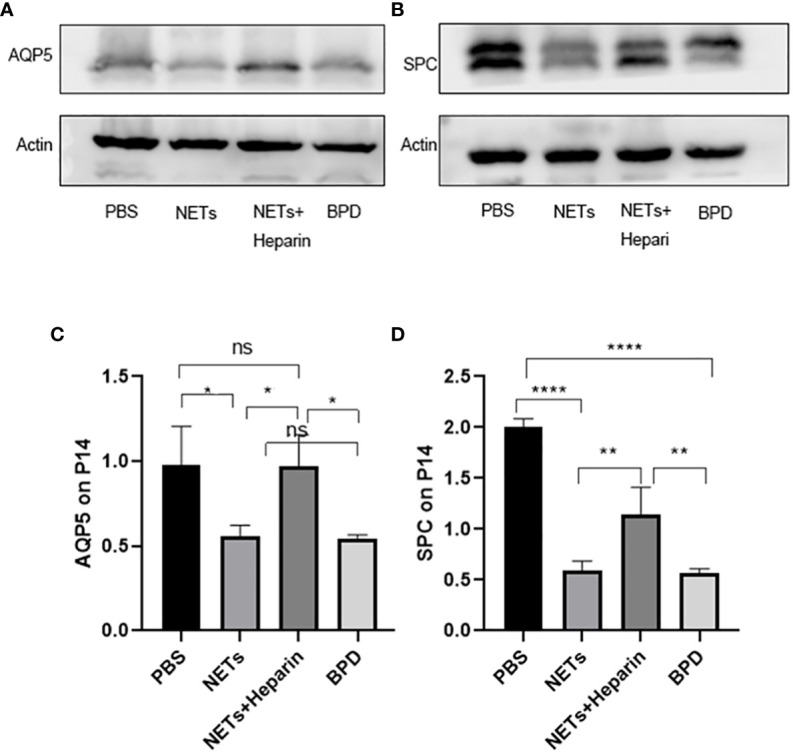
The effect of NETs on alveolar differentiation and development was determined by Western blotting. **(A, B)**. The expression of AQP5 and SPC was determined by Western blotting. **(C, D)** Greyscale value of the Western blot results. Statistical analysis was performed by one-way ANOVA with a *post hoc* Tukey’s test **(C, D)**. n=3, ****p ≤ 0,001, **p ≤ 0.005, *p < 0.05; n.s, not significant. one-way ANOVA).

Compared to that in the PBS control group, AQP5 expression was lower in the NETs group, as shown by qPCR (P<0.05, one-way ANOVA) (**Figure 8**). Western blotting showed that the levels of AQP5 and SPC in the NET group were significantly lower than those in the PBS control group and NET+heparin group (P<0.05 one-way ANOVA). These results demonstrate that NETs impaired pulmonary epithelial cell development and led to BPD-like changes, while heparin improved alveolar development and alleviated pulmonary BPD-like changes (P<0.05 one-way ANOVA) (**Figure 9**).

### 3.6 Effects of NETs on target genes of the WNT/β-catenin pathways

**Figure 10:**
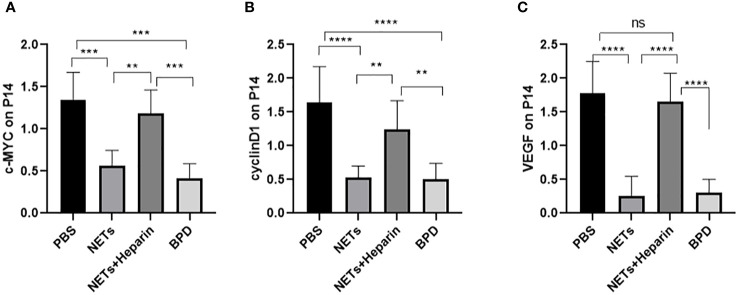
The expression of c-MYC, cyclin D, and VEGF in each group. **(A–C)** Relative gene expression of c-MYC, cyclin D1, and VEGF. Statistical analysis was performed by one-way ANOVA with a *post hoc* Tukey’s test (n=5, **≤0.005,***p ≤ 0.0008, ****p ≤ 0,0001. n.s, not significant).

**Figure 11:**
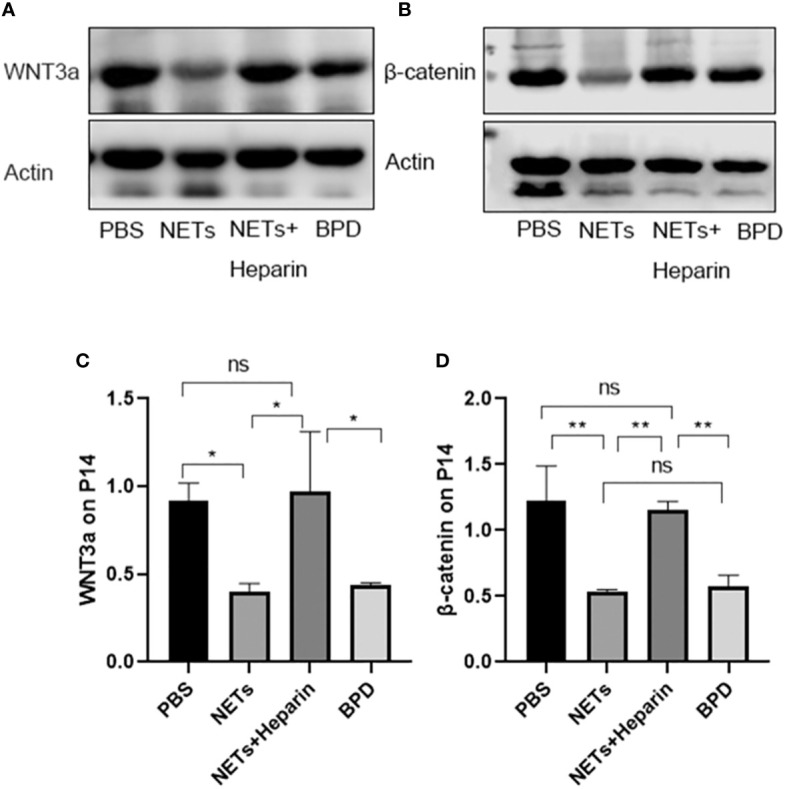
Expression of WNT3a and β-catenin. **(A, B)**. The expression of WNT3a and β-catenin was determined by Western blotting. **(C, D)**. The greyscale values for WNT3a and β-catenin. **(C)**. Statistical analysis was performed by one-way ANOVA with a *post hoc* Tukey’s test (n=3, *p < 0.05; **p ≤ 0,01; n.s, not significant).

The WNT/β-catenin pathway is the most classic signalling pathway that affects lung development, and c-MYC, cyclin D1, and VEGF are important target genes of this pathway. To determine whether NETs affect lung development *via* this pathway, we examined the expression of important target genes of this pathway in each group (**Figure 10**). We found that the expression of c-MYC, cyclin D1, and VEGF was significantly lower in the NET group than in the PBS control group, as determined by RT−PCR. The NETs + heparin group had significantly higher expression than the NET group (P<0.05 one-way ANOVA) (**Figure 11**).

We also used Western blotting to evaluate the effects of NETs on the expression of important WNT/β-catenin proteins. We found that the levels of WNT3a and β-catenin in the NET group and hyperoxia-induced lung injury group were significantly lower than those in the PBS control group.

NETs influence downstream target gene and protein expression through the WNT/β-catenin pathway, leading to the development of BPD in preterm infants.

## 4 Discussion

BPD is a severe and sometimes lethal chronic lung disease in prematurely born neonates, especially those with very low birth weight (VLBW) and extremely low birth weight (ELBW) (Davidson and Berkelhamer, 2017). Pulmonary immaturity, infection, hyperoxia, and other adverse conditions can lead to lung damage in preterm newborns, resulting in pulmonary fibrosis or the obstruction of alveolar and pulmonary vascular development. The exacerbation of VLBW and ELBW in newborns leads to a corresponding increase in the incidence of BPD, despite the use of protective strategies such as prenatal steroid administration, postnatal surfactant supplementation, and safer ventilator measures. Extremely high mortality, chronic respiratory dysfunction, neurodevelopmental difficulties, prolonged hospitalization, large expenditures, and significant social and household burdens are all consequences of severe BPD. VLBW and ELBW infants are mostly born in the tubular or cystic stages of lung development. Normal lung development in these stages might be blocked due to various adverse factors, resulting in BPD. However, the pathogenesis of BPD is still unclear.

Neutrophils are the first line of defence against invading pathogens (Brinkmann et al., 2004). Neutrophils can form NETs to remove extracellular pathogens (Rosales et al., 2016) and are involved in the development of a variety of inflammatory diseases of the lungs, such as COPD and acute lung injury (Jorch and Kubes, 2017; Söderberg and Segelmark, 2018). In addition to bacteria-induced NETs (Chen et al., 2021), viruses (Saitoh et al., 2012), cholesterol and urate crystals (Chen et al., 2021), cytokines (Warnatsch et al., 2015), calcium ionophores (Tatsiy and McDonald, 2018), bacterial lipopolysaccharides (Kenny et al., 2017), and Fobosol-12-Myriam-13-acetate (Pieterse et al., 2016) can also induce NETs production. Therefore, in this study, PMA was used to induce neutrophils obtained from human peripheral blood, and NETs were administered to mice. Heparin binds to extracellular histones and reduces the activity of NETs, thereby affecting blood clotting and infection-related vascular function (Keshari et al., 2013). Anti-histone antibodies and heparin can reduce lung damage by inhibiting NET formation. Therefore, heparin was used as an inhibitor of NETs in this study. Similar to the conclusions of other researchers, our analysis of children with and without BPD found that the levels of NETs in children with BPD were significantly higher than those in children without BPD. In animal experiments, it was shown that the levels of NETs in the hyperoxia-induced BPD group were higher than those in the PBS control group, which showed that NETs were closely associated with BPD.

AQP1 and AQP5 play important roles in lung pathophysiology and disease, including chronic and acute lung injury, COPD (COPD), other inflammatory lung diseases, and lung cancer (Gardiner and Andrews, 2012). Rodent studies showed that four AQPs are widely distributed in airway epithelial cells, alveolar epithelial cells, associated microvascular endothelial cells, and submucosal glands (Yadav et al., 2020). AQP5 is barely expressed at birth and gradually increases after birth until adulthood (Verkman, 2007). SP-A, SP-B, SP-C, and SP-D 4 are pulmonary surfactant proteins that work together to maintain the stability of pulmonary surfactants and improve lung compliance to prevent lung tissue collapse. SP can regulate PS secretion and clearance by regulating the level of PS in the alveoli to maintain alveolar stability of the environment. SPC can also stabilize the effect of surfactant membranes during respiratory motion by increasing the absorption of AECII. Our study showed that AQP5 and SPC levels were decreased in the NETs group compared to the PBS control group, but there was no significant difference in the hyperoxia-induced lung injury group.

Based on our findings, NETs interfered with alveolar epithelial cell differentiation and function, as well as lung development.

The WNT/β-catenin pathway is one of the most important and classical pathways involved in alveolar development. An animal study showed that the WNT/β-catenin pathway could influence lung differentiation and development, particularly during the cystic stage, resulting in clinical BPD-like changes (Jin et al., 2020). Moreover, the classical WNT/β-catenin pathway regulates inflammatory cytokines (TNF, IL-1, IL-6, IL-8, and IL-15), which play important roles in reducing lung inflammation and pulmonary fibrosis and are linked to the pathogenesis of BPD (Hu et al., 2020). Furthermore, the classical WNT/β-catenin pathway is a vital regenerative pathway for chronic lung disease, as it is required for the differentiation and survival of the alveolar epithelium and progenitor cells after lung injury. If this pathway is inhibited, epithelial cell repair in preterm infants with COPD and asthma is blocked (Raslan and Yoon, 2020). Thus, the classical WNT/β-catenin signalling pathway might play an important role in the development of BPD. When the Wnt ligand is present, Wnt binds to the coreceptor low-density lipoprotein receptor-related protein 5/6 (LRP5/6) and the cell membrane receptor Frizzled, and destroys the degradation complex; β-catenin is not phosphorylated, is displaced to the nucleus, and binds to the transcription factor TCF/LEF to initiate the transcription of downstream target genes (mainly associated with lung development: VEGF, c-MYC, cyclin D1, etc.), promoting cell proliferation, differentiation and maturation to exert its physiological effects. Compared to those in the control group, the levels of cyclin D1, c-MYC, and VEGF were decreased in the NETs group and the hyperoxia-induced lung injury group. This finding suggests that the Wnt/β-catenin pathway is inhibited by NETs, while the levels of downstream target genes can be moderately upregulated by the NETs inhibitor heparin. Further studies showed that the levels of WNT3a and β-catenin in the NETs group were significantly lower than those in the PBS control group. Therefore, NETs inhibit this pathway and decrease the expression of downstream target genes. Heparin can antagonize the inhibition of WNT3a and β-catenin by NETs, thereby promoting the expression of downstream target genes and proteins. NETs inhibitors are regarded as a novel approach to treating related diseases Tsai and Hwang (2015), and in this study, we demonstrated that 250 U/kg heparin could inhibit NETs in an animal model, mitigating BPD-like changes in mouse lungs. As a result, heparin, which is a NET inhibitor, could be a potential treatment for BPD.

We confirmed the correlation between NETs and BPD in clinical specimens. Furthermore, in the preterm mouse model, we found that NETs inhibited the expression of WNT3a, β-catenin, and downstream target genes in the WNT/β-catenin pathway, impairing lung development and promoting the occurrence of BPD. NETs might be a novel target in the prevention and treatment of BPD in preterm infants, and NET inhibitors might serve as potential therapeutic agents for BPD.

## Data availability statement

The original contributions presented in the study are included in the article/**Supplementary Material**. Further inquiries can be directed to the corresponding author.

## Ethics statement

The studies involving human participants were reviewed and approved by Ethics Committee of Children’s Hospital Affiliated to Chongqing Medical University. Written informed consent to participate in this study was provided by the participants’ legal guardian/next of kin. The animal study was reviewed and approved by Experimental Animal Ethics Committee of Chongqing Medical University.

## Author contributions

All authors participated in the collection of clinical specimens. MZ, JJ, WL and WZ contributed to the writing of individual sections. LS was responsible for the whole manuscript and provided the final version for review. MZ and LS proofread the final version. All authors contributed to the article and approved the submitted version.
