# Supplementary material for: Neutrophil extracellular traps promote bronchopulmonary dysplasia-like injury in neonatal mice via the WNT/β-catenin pathway
Source: Front Cell Infect Microbiol. 2023 Apr 27;13:1126516. doi: 10.3389/fcimb.2023.1126516 (PMC10174450; doi:10.3389/fcimb.2023.1126516)
Supplement: Supplementary file 12 [file Table_12.docx]

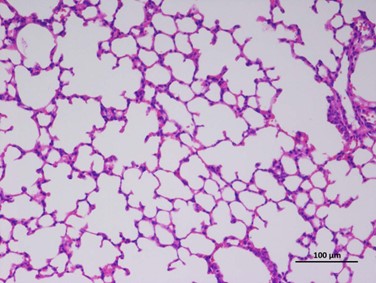


Fig1:H&E assessment of lung tissue in NETs+Heparin group(×200)


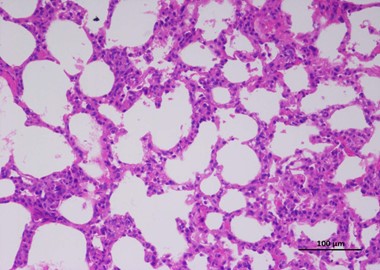


Fig2:H&E assessment of lung tissue in BPD group(×200)


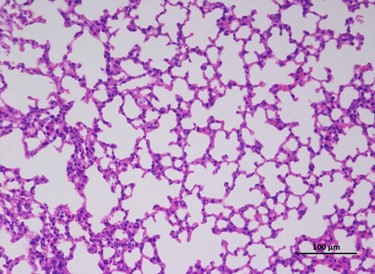


Fig3：H&E assessment of lung tissue in PBS group (×200)


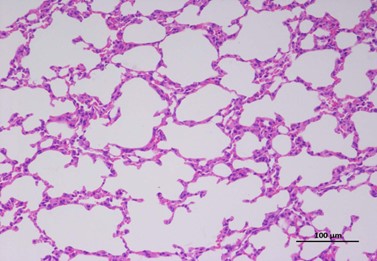


Fig4：H&E assessment of lung tissue in NETs group (×200)


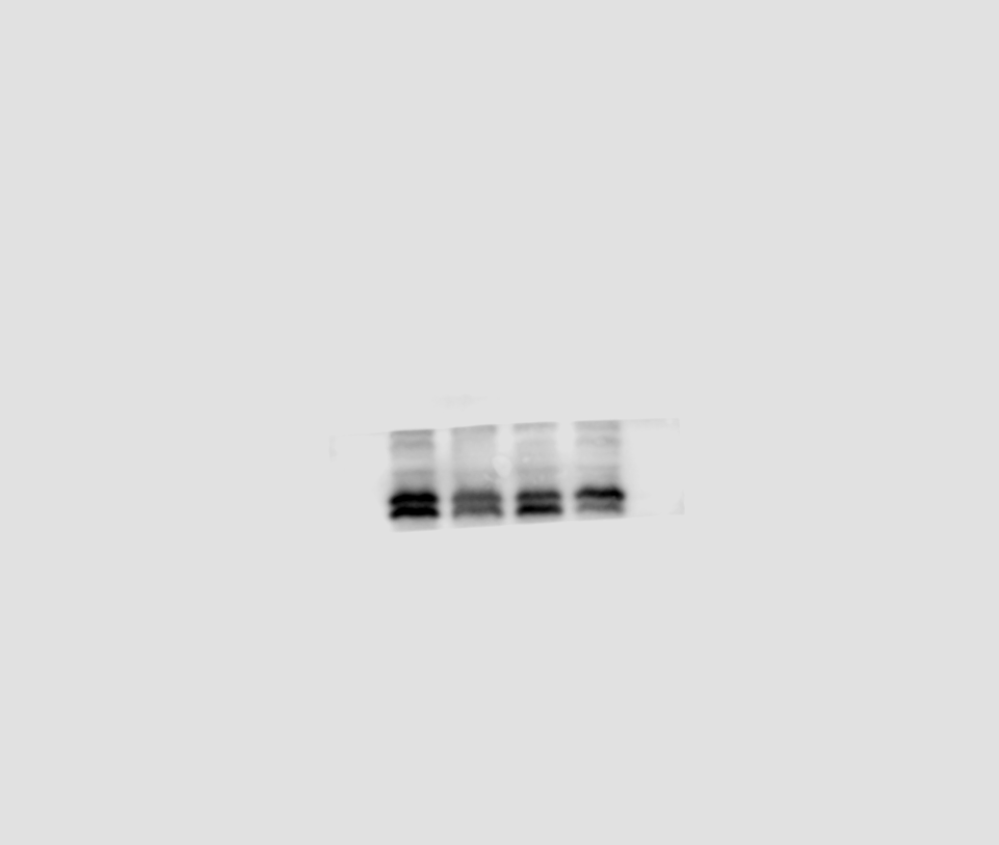


Fig5：The expression of SPC was determined by Western blotting.


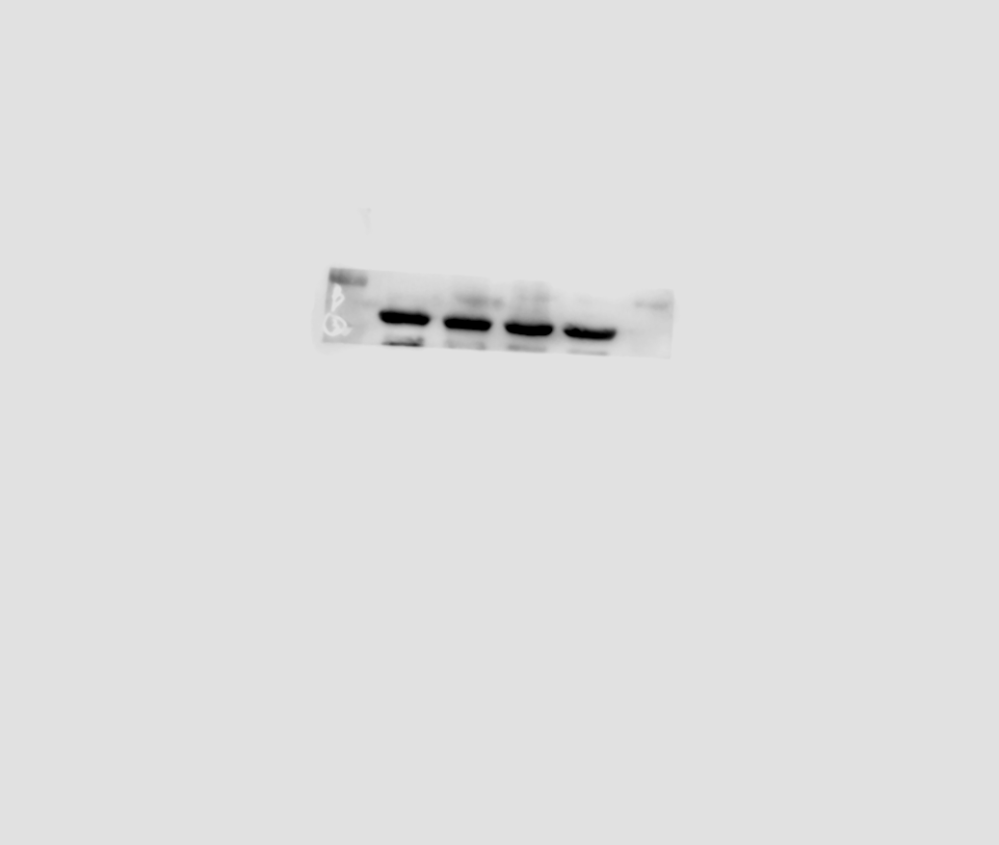


Fig6：The expression of Actin was determined by Western blotting.


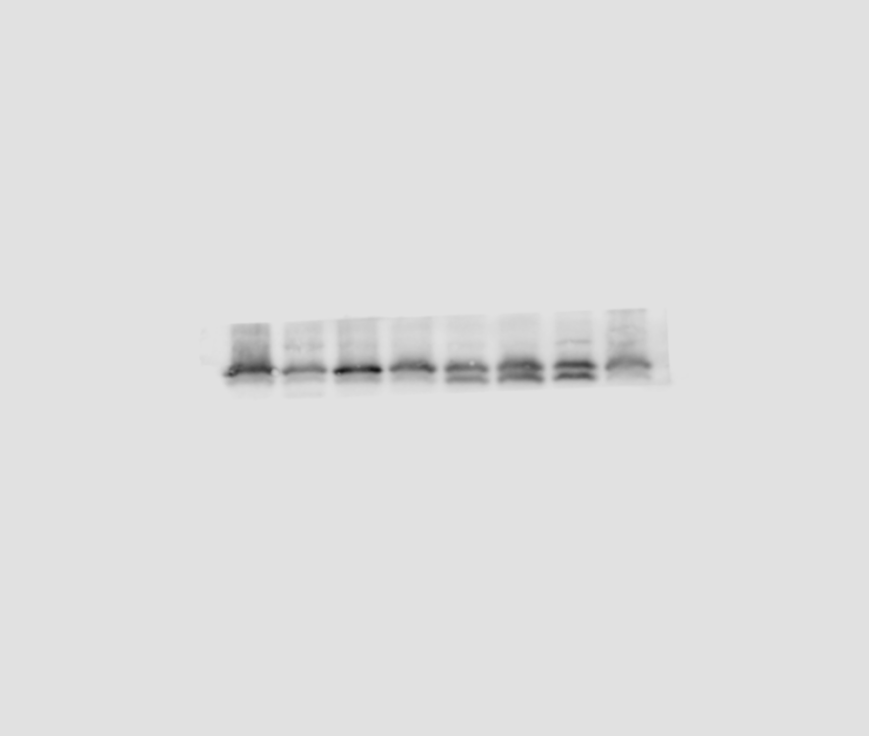


Fig7：The expression of AQP5 was determined by Western blotting.


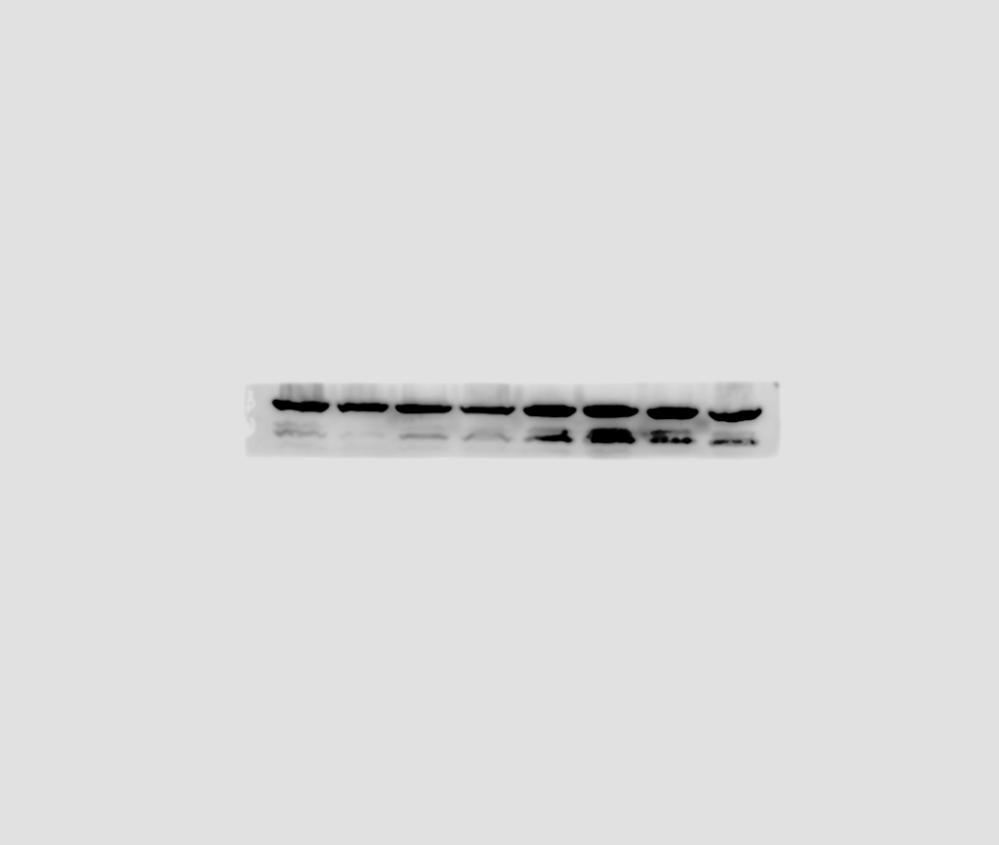


Fig8：The expression of Actin was determined by Western blotting.


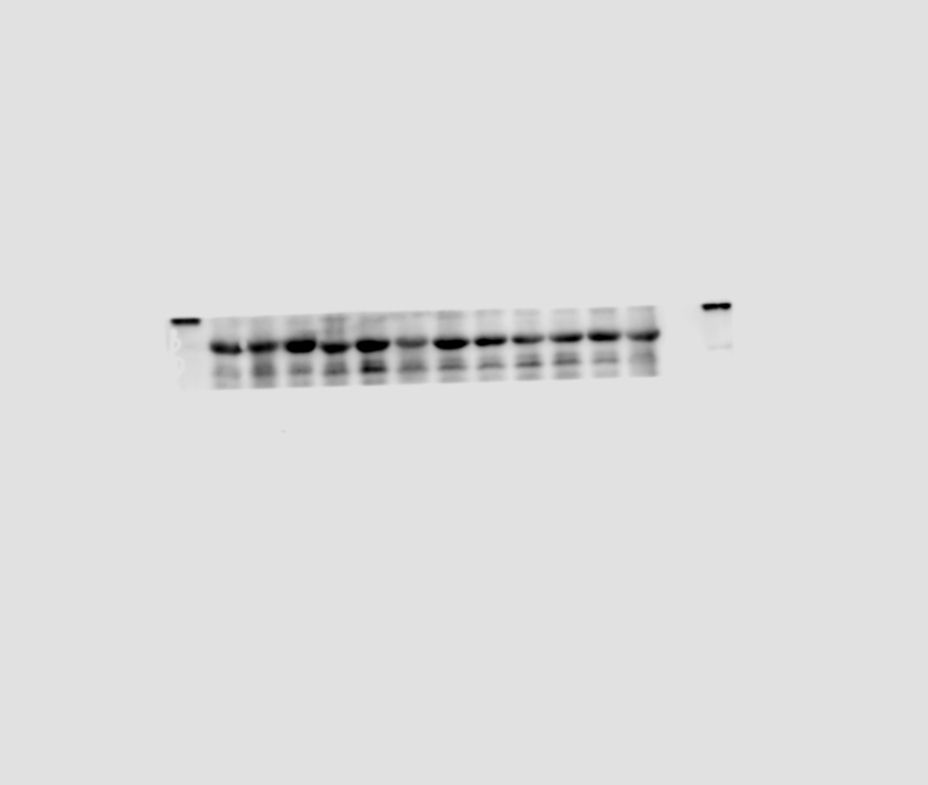


Fig9：The expression of WNT3a was determined by Western blotting.


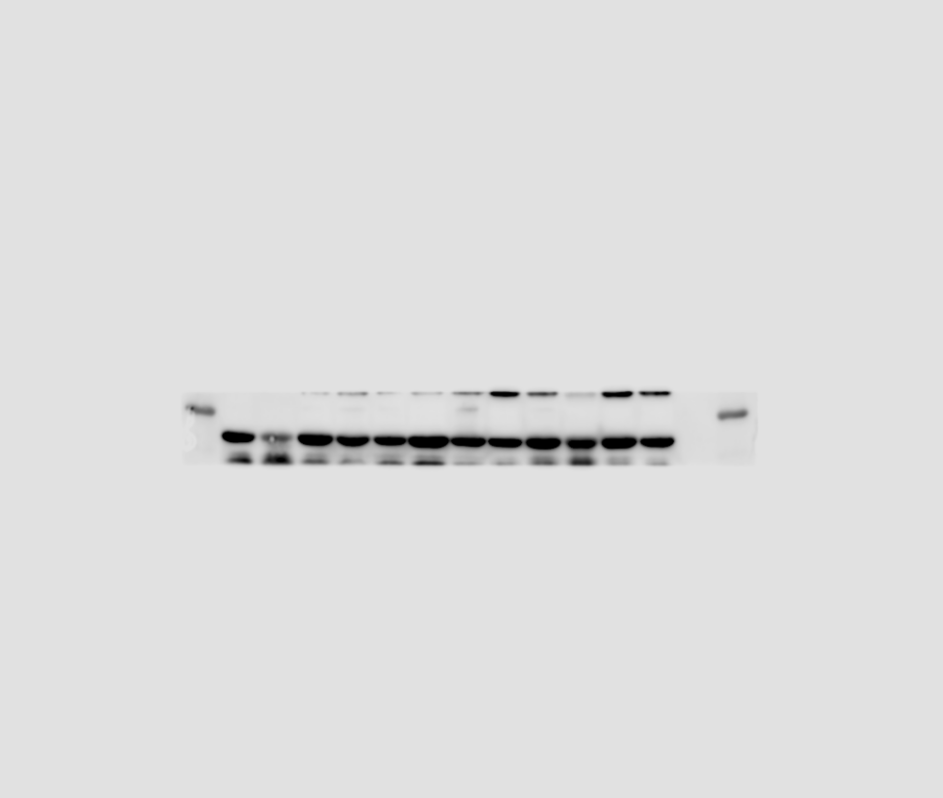


Fig10：The expression of Actin was determined by Western blotting.


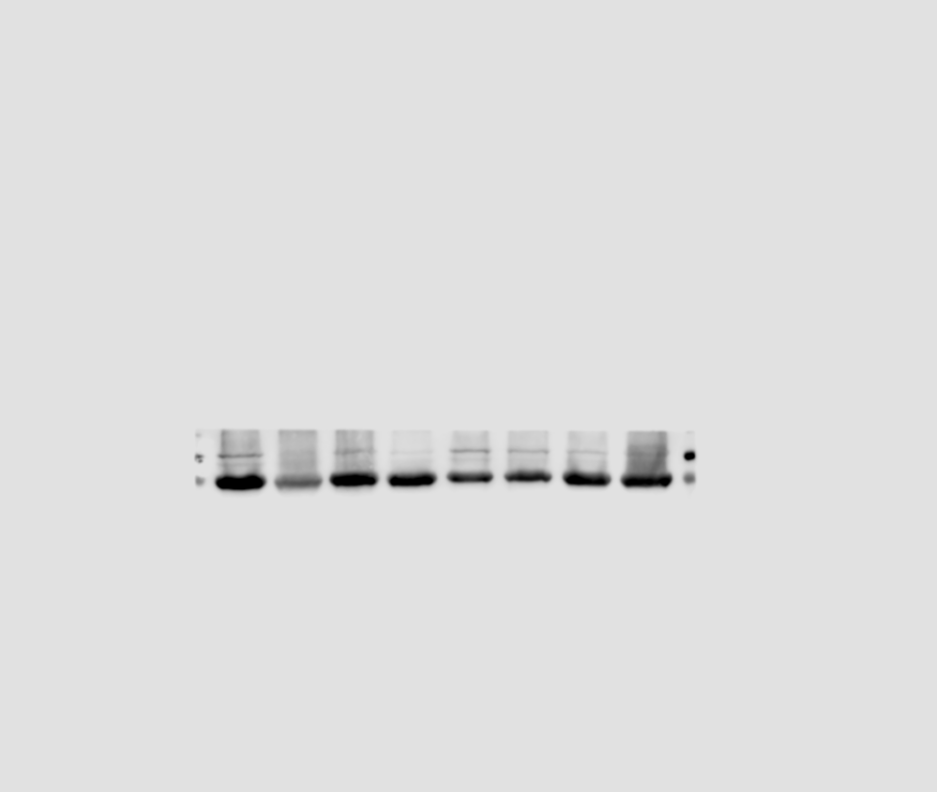


Fig11：The expression of β-catenin was determined by Western blotting.


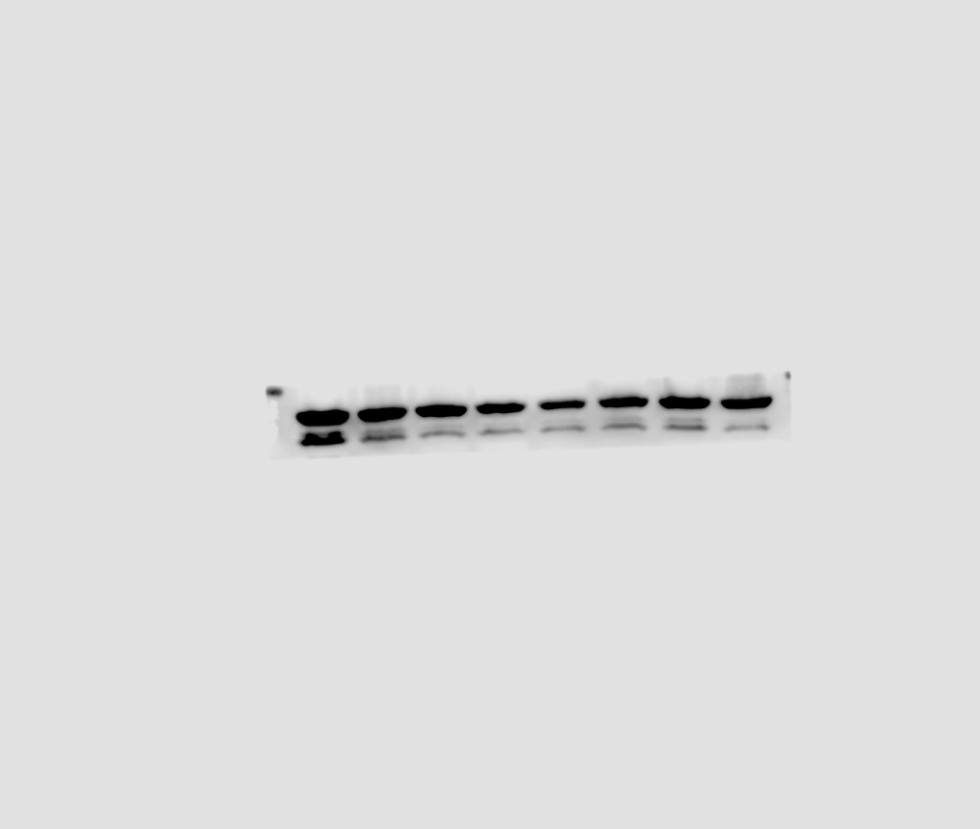


Fig12：The expression of Actin was determined by Western blotting.


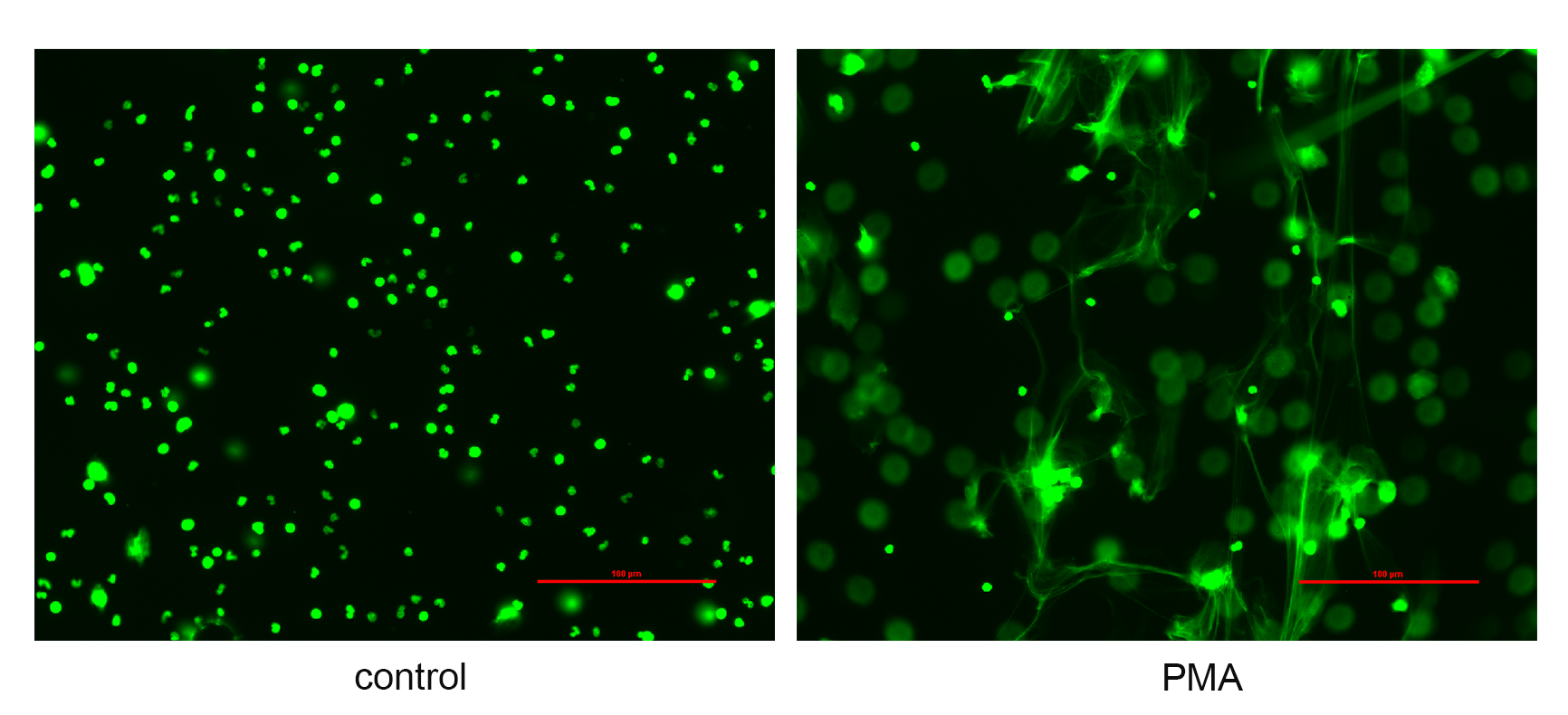


Fig13：NETs were induced by PMA (Fluorescence microscopy) (× 200); bar: 100 µm.


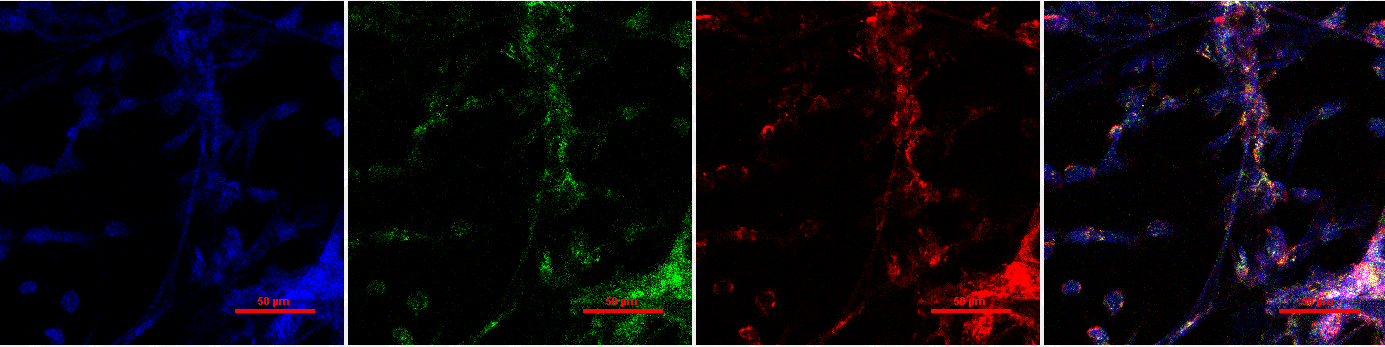


Fig14：NETs were induced by PMA (immunofluorescence) (× 400); bar: 50 µm.


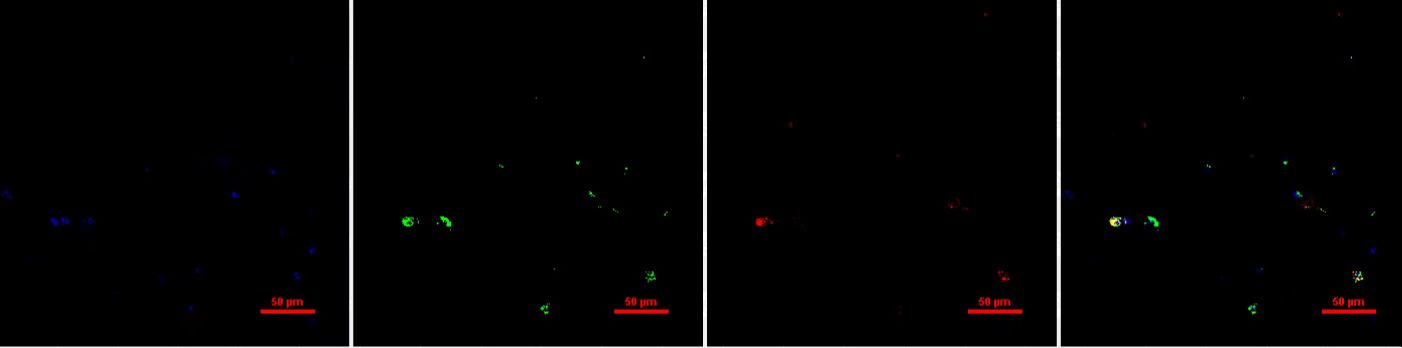


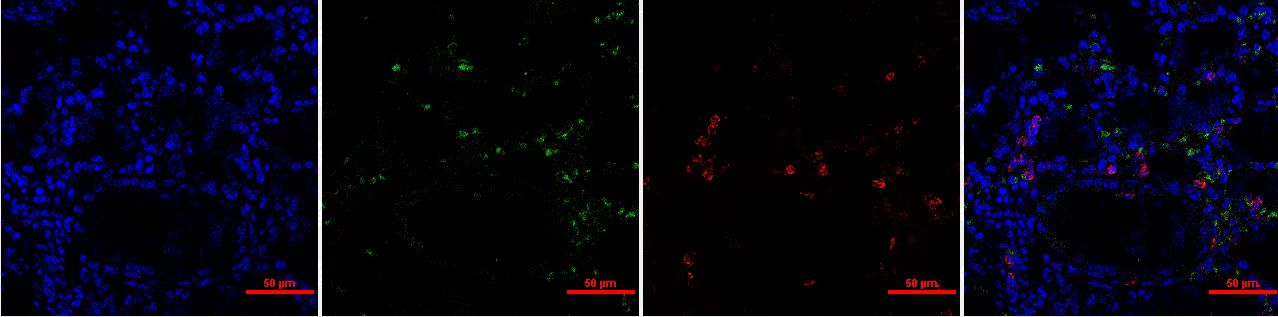


Fig15：Comparison of the immunofluorescence of NETs in the PBS control group and hyperoxia-induced lung injury group (× 400); bar: 50 µm.
